# Progressive spastic tetraplegia and axial hypotonia (STAHP) due to SOD1 deficiency: is it really a new entity?

**DOI:** 10.1186/s13023-021-01993-0

**Published:** 2021-08-11

**Authors:** Paulo Victor Sgobbi de Souza, Wladimir Bocca Vieira de Rezende Pinto, Igor Braga Farias, Bruno de Mattos Lombardi Badia, Icaro França Navarro Pinto, Gustavo Carvalho Costa, Carolina Maria Marin, Ana Carolina dos Santos Jorge, Emília Correia Souto, Paulo de Lima Serrano, Roberta Ismael Lacerda Machado, Marco Antônio Troccoli Chieia, Enrico Bertini, Acary Souza Bulle Oliveira

**Affiliations:** 1 Department of Neurology and Neurosurgery, Federal University of São Paulo (UNIFESP), Embaú Street, 67, Vila Clementino, São Paulo, SP 04039-060 Brazil; 2 Unit of Neuromuscular and Neurodegenerative Disorders, Bambino Gesù Children’s Research Hospital, IRCCS, Rome, Italy

**Keywords:** Amyotrophic lateral sclerosis, Hypotonia, Progressive spastic tetraplegia, *SOD1* mutation, SOD1 deficiency, Childhood neurodegenerative disorder, Motor neuron disease, Neuromuscular disorders

## Abstract

**Background:**

Amyotrophic Lateral Sclerosis (ALS) is a rare, progressive, and fatal neurodegenerative disease due to upper and lower motor neuron involvement with symptoms classically occurring in adulthood with an increasing recognition of juvenile presentations and childhood neurodegenerative disorders caused by genetic variants in genes related to Amyotrophic Lateral Sclerosis. The main objective of this study is detail clinical, radiological, neurophysiological, and genetic findings of a Brazilian cohort of patients with a recent described condition known as Spastic Tetraplegia and Axial Hypotonia (STAHP) due to SOD1 deficiency and compare with other cases described in the literature and discuss whether the clinical picture related to SOD1 protein deficiency is a new entity or may be represent a very early-onset form of Amyotrophic Lateral Sclerosis.

**Methods:**

We conducted a case series report which included retrospective data from five Brazilian patients with SOD1 protein deficiency of a Brazilian reference center for Neuromuscular Disorders. Clinical data were obtained from a review of the medical records and descriptive statistics and variables were summarized using counts and percentages of the total population.

**Results:**

All 5 patients presented with a childhood-onset neurodegenerative disorders characterized by spastic tetraplegia with axial hypotonia in all cases, with gestational history showing polyhydramnios in 4/5 and intrauterine growth restriction in 3/5 patients, with most patients initially presenting a normal motor development until the six month of life or during the first year followed by a rapidly progressive motor decline with severe dysphagia and respiratory insufficiency in all patients accompanied by cognitive impairment in 3/5 patients. All patients were homozygous for the c.335dupG (p.Cys112Trpfs*11) mutation in the *SOD1* gene with completely decreased enzyme activity.

**Conclusions:**

This case series is the biggest data collection of the new recent clinical entity described as Spastic Tetraplegia and Axial Hypotonia (STAHP) due to SOD1 deficiency.

## Background

The Superoxide Dismutase (SODs) enzymes are a class of enzymes important to catalyze the inactivation of superoxide radicals (O_2_^−^) into oxygen and hydrogen peroxide and provide an important cellular antioxidant defense through redox reactions. In humans there are three different isoforms of SODs: human Cu–Zn SOD (SOD1), the mitochondrial MnSOD (SOD2) and the extracellular Cu–Zn SOD (SOD3), and impairment of their antioxidant function or overactivity due to gain of function molecular mechanisms, represents a major pathophysiology role in the development of human neurodegenerative disorders (primarily, Amyotrophic Lateral Sclerosis) and cancer linked to SOD1 abnormalities [1, 2].

The human Cu–Zn SOD (SOD1) is a metalloenzyme with 153 amino acids residues encoded by *SOD1* gene localized on chromosome locus 21q.22.1 that contains five exons which are separated by four introns [1, 2]. The SOD1 protein is an active dimer Cu–Zn enzyme composed of eight antiparallel beta strands and two metal atoms that catalyze the toxic superoxide (O_2_^−^) into oxygen (O_2_) and hydrogen peroxide (H_2_O_2_), with new functions reported such as activation of nuclear gene transcription following exposure to oxidative stress, regulation of RNA metabolism, and modulation of glucose metabolism to interact with different molecules (e.g., other proteins, membranes, and nucleic acids) [1, 2].

**Fig. 1:**
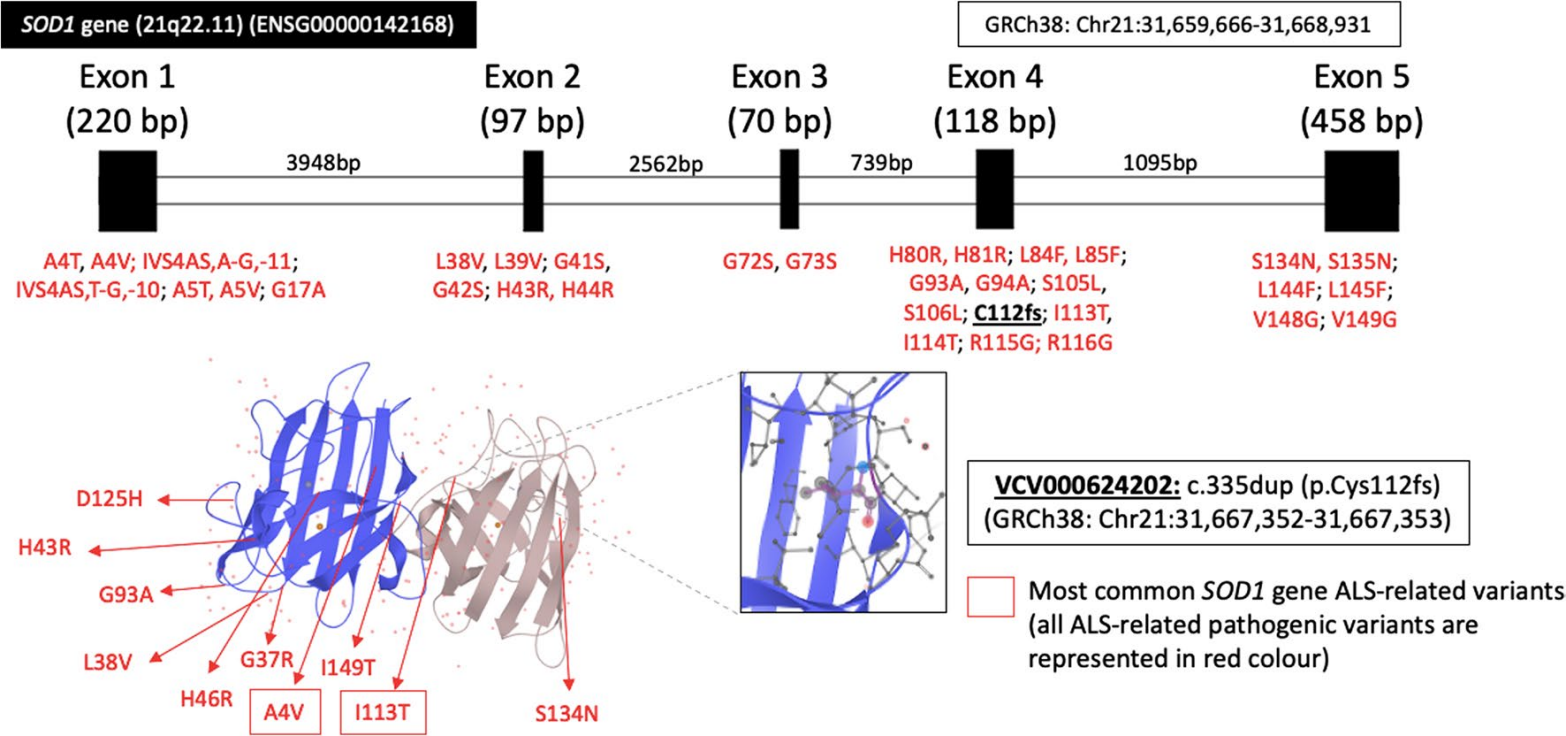
SOD1 gene. Gene structure and their known variants

In 1993, mutations in *SOD1* were associated to familial autosomal dominant Amyotrophic Lateral Sclerosis (ALS), and nowadays they represent the second most common cause of sporadic (up to 7% of cases) and familial ALS giving rise to up to 20% of cases with an autosomal dominant or recessive inheritance, with more than 150 variants (Fig. 1) identified in different geographic populations [2–4].

Recently, Andersen et al. [5] and Park et al. [6] reported independently two Afghan patients with a severe neuromuscular disorder characterized by progressive motor neuron disease with hypotonia, spastic tetraplegia and loss of motor function due to *SOD1* homozygous biallelic loss of function truncating mutations with no family history of ALS and concluded that this disorder is distinct from *SOD1* related ALS and constitutes a new clinical entity [5, 6].

In this study, we present detailed clinical, genetic, radiological, and neurophysiological features of 5 Brazilian patients with SOD1 deficiency and compare our patients with the literature review to better delineate clinical features of this new clinical entity.

## Methods

This study reviewed medical records of 5 Brazilian patients from two non-related Lebanese families with SOD1 deficiency due to homozygous mutation in *SOD1* gene followed at the Universidade Federal de São Paulo. All patients’ parents gave written informed consent for participation in the study and for this publication, and study procedures were approved by institutional ethics committees (CAAE: 93830518.5.0000.5505).

All 5 patients and their parents are alive and were invited to a single medical interview for review of medical data, clinical and neurological examination, and referral for brain MRI and neurophysiological studies.

Brain MRI was performed in all subjects with a 1.5 T MRI system (Magnetom Sonata [Maestro Class]—Siemens AG, Medical Solutions, Erlangen, Germany) using an eight- channel head coil. The following sequences were acquired: (1) sagittal images (T1-weighted spin echo); (2) coronal images [(T2-weighted fast spin echo, T2-weighted fluid-attenuated inversion recovery (FLAIR), T1-weighted inversion recovery)]; and (3) axial images (T2- weighted FLAIR, T2-weighted gradient echo).

Muscle MRI study of the lower limbs were performed in a 1,5 T scanner with 4-channel phased-array coil (Magnetom Avanto; Siemens, Munich, Germany) using a standardized protocol with axial images (T1-weighted spin-echo [repetition time (TR)/echo time (TE) 335/19 ms, slice thickness 7 mm, matrix 360–464 × 512], short tau inversion recovery [TR/TE 3,650/52 ms, inversion time 160 ms, slice thickness 7 mm, matrix 480–576 × 512]).

All patients underwent Nerve Conduction Studies (NCS) and electromyography (EMG) at least one time in the period of the study in the same laboratory with NCS of bilateral median, ulnar, peroneal, tibial, and sural nerves, and needle EMG in proximal and distal muscles of the upper and lower limbs and paravertebral regions using methods previously described [7].

Whole exome sequencing (WES), clinical exome, and a targeted gene sequencing panel for Amyotrophic Lateral Sclerosis were performed in all five patients and their parents, respectively. WES was performed using SureSelect_AllExons_V5_ gene (Agilent Technologies, Santa Clara, CA, USA), clinical exome with TruSight One (Illumina, San Diego, CA, USA), and the target-panel with SureSelect Human All Exon V6 (Agilent Technologies) following the manufacturers’ instructions. The sequencing was analyzed according to the American College of Medical Genetics and Genomics recommendations. In silico variant evaluation was carried out using the prediction software MutationTaster as well as PolyPhen-2 [8, 9].

The functional activity of superoxide dismutase activity was performed in erythrocytes using a spectrophotometric approaching according to a previous published protocol [6, 10, 11].

Muscle biopsy was performed in all patients using an open technique in the deltoid muscle. Muscle samples stored at a temperature of − 80 °C were cut into fragments with a depth of 6–8 μm and mounted on slides, which were subsequently stained with hematoxylin and eosin, modified Gömöri trichrome, periodic acid-Schiff, Oil-red O, and submitted to histoenzymology for reduced nicotinamide adenine tetrazolium reductase, cytochrome C oxidase (COX), succinate dehydrogenase, and ATPase at two different pH values: 9.4 and 4.6. Respiratory chain enzyme activities were measured spectrophotometrically, as previously described in muscle specimens [12].

All patients were submitted for blood tests including complete blood counts, creatinine and urea, serum creatine kinase (CPK), liver enzymes, thyroid function (TSH and free thyroxine), lipid profile, serum lactate, plasma acylcarnitine profile and urinary organic acids.

All patients were evaluated with the following functional assessment scales: (1) Hammersmith Infant Neurological Examination – Section 2 – Motor Milestones (HINE-2); (2) Children’s Hospital of Philadelphia Infant Test of Neuromuscular Disorder (CHOP-INTEND) and (3) Expanded Hammersmith Functional Motor Scale for SMA (HFMSE).

Descriptive statistics was applied, and variables were summarized using counts and percentages of the total population. Variables were summarized using descriptive statistics including mean, median, ranges, percentages and/or frequencies.

## Results

**Fig. 2:**
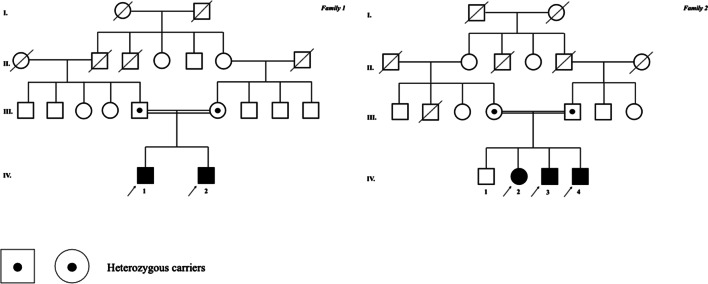
Pedigree. The pedigree of the families described in this article is fully represented and the index patients are indicated by a full arrow

The main clinical data are described in Table 1. The pedigrees of the two reported families are presented in Fig. 2.

**Table 1:** Detailed clinical features

	Patient 1	Patient 2	Patient 3	Patient 4	Patient 5
*General features*					
Current age (year)	3 y	2 y	7 y	5 y	2 y
Age at onset (month)	7 m	9 m	12 m	15 m	12 m
Gender	M	M	F	M	M
Consanguinity	Yes	Yes	Yes	Yes	Yes
Ancestry	Lebanese	Lebanese	Lebanese	Lebanese	Lebanese
*Gestational and neonatal history*					
Polyhydramnios	Yes	Yes	No	Yes	Yes
Reduced fetal movements	No	Yes	No	Yes	No
Intrauterine growth restriction	Yes	No	No	Yes	Yes
Premature labor	Yes	No	No	Yes	No
Apgar score (1’/5’)	5/7	7/9	9/10	3/5	7/9
Hypotonia (at birth)	Moderate	Mild	No	Severe	No
Bulbar dysfunction (at birth)	No	Dysphagia	No	Dysphagia	No
Respiratory distress (at birth)	No	No	No	Yes	No
Congenital dislocation of hip	Bilateral	Bilateral	No	Bilateral	No
*Clinical features*					
Spastic tetraplegia	Yes	Yes	Yes	Yes	Yes
Axial hypotonia	Yes	Yes	Yes	Yes	Yes
Cervical weakness	Yes	Yes	No	Yes	No
Proximal muscle weakness	Yes	Yes	Yes	Yes	Yes
Distal muscle weakness	Yes	Yes	Yes	Yes	Yes

Muscle atrophy	Proximal/tongue	Proximal/tongue	Proximal/tongue	Proximal/distal/tongue	Proximal/distal/tongue
Fasciculations	T/C/LL	T/C/UL/LL	T/C/UL/LL	T/C/UL/LL	T/LL
Facial myokymia	Yes	No	Yes	Yes	No
Facial weakness	Yes	Yes	Yes	Yes	No
Dysphagia	Yes	Yes	Yes	Yes	Yes
Gastrostomy feed	Yes	Yes	Yes	Yes	Yes
Tracheostomy	No	Yes	Yes	Yes	No
Ventilatory support	Non-invasive	Invasive	Invasive	Invasive	Non-invasive
Brisk tendon reflexes	Yes	Yes	Yes	Yes	Yes
Babinski sign	Yes	Yes	Yes	Yes	Yes
Scoliosis	No	No	Yes	Yes	Yes
Dysarthria	Yes	Yes	Yes	Yes	Yes
Language defects	Yes	Yes	Yes	No	No
Cognitive impairment	Severe	Moderate	Mild	No	No
Eye movements	Oculomotor apraxia	Oculomotor apraxia	Normal	Oculomotor apraxia	Normal
Optical fundoscopy	Optic atrophy	Normal	Normal	Optic atrophy	Normal
Epilepsy	No	Yes	No	No	No
Brainstem auditory evoked potentials	Normal	Normal	Normal	Normal	Normal
Microcephaly	Yes	Yes	No	No	No
Short Stature	Yes	Yes	No	Yes	No
Constipation	Yes	Yes	Yes	Yes	Yes
Best motor milestone achieved	Head control	Sit unassisted	Walk independently	Walk independently	Sit unassisted
Ambulatory status	Wheelchair	Wheelchair	Wheelchair	Wheelchair	Wheelchair
CPK (UI/L)	520	610	780	630	400

C: Cervical. F: female. LL: lower limb. M: male. T: tongue. UL: upper limb.

### Case report

#### Family 1

Patient 1. The patient is a Brazilian 3-year-old boy, first child of consanguineous parents (first degree cousins) of Lebanese origin. During pregnancy, polyhydramnios was noted. The patient was born at 34 weeks of pregnancy via Caesarean section due to maternal preeclampsia. The Apgar score at 1 and 5 min was 5 and 7, respectively. At birth patient was noted to have hypotonia and bilateral congenital dislocation of the hip, without respiratory distress or bulbar dysfunction. The parents reported normal development delay until 7 months when the patient was still able to maintain head control followed by progressive psychomotor decline with inability to sit, to roll, and standing. At first clinical evaluation patient presented a severe spastic tetraplegia, pyramidal features such as Babinski sign and bilateral Achilles’ clonus, axial hypotonia with cervical weakness, proximal muscle atrophy in the upper and lower limbs, fasciculations of the tongue, cervical region, and quadriceps femoris muscle groups, facial myokymia, and severe cognitive impairment.

Patient 2. This patient is a 2-year-old boy, brother of patient 1, with a pregnancy history of polyhydramnios. The patient was born at 39 weeks of pregnancy by Caesarean delivery due to fetal malpresentation. The Apgar score at 1 and 5 min was 7 and 9, respectively. At birth hypotonia was noted with transient bulbar dysfunction and dysphagia. The parents reported normal development until the age of 9 months when the patient was able to maintain head control, and was stably sitting, but was thereafter followed by progressive psychomotor decline and bulbar dysfunction. At first clinical evaluation patient presented a severe spastic tetraplegia, Babinski sign, axial hypotonia with cervical weakness, proximal muscle atrophy in the upper and lower limbs and fasciculations at tongue, cervical region, biceps brachii, quadriceps femoris and tibialis anterior muscles group and cognitive impairment without language development.

#### Family 2

Patient 3. The patient is a Brazilian 7-year-old girl, second child of consanguineous parents (first degree cousins) of Lebanese origin, who after 1 year of age, developed over 2 years progressive manifestations of psychomotor deterioration, chronic respiratory insufficiency with tracheostomy and invasive mechanical ventilation after an episode of pneumonia, and swallowing difficulties requiring gastrostomy feed. Her previous motor milestones were unremarkable with the ability to walk independently at 1 year of age. Previous medical history exhibited an uncomplicated pregnancy with a natural labor at 39 weeks of pregnancy without neonatal hypotonia or events suggestive of possible hypoxic-ischemic encephalopathy. Neurological examination revealed mild spastic tetraplegia, proximal muscle weakness of upper and lower limbs, axial hypotonia, diffuse fasciculations, facial diparesis, tongue atrophy, dysarthria, scoliosis, and language impairment.

Patient 4**.** The patient is a 5-year-old boy, brother of patient 3, who presented with 3-year history of progressive psychomotor regression, chronic respiratory insufficiency, and dysphagia. His previous motor milestones were unremarkable with the ability to walk independently at 15 months of age. During pregnancy, polyhydramnios, intrauterine growth restriction and reduced fetal movements was noted. The patient was born at 36 weeks of pregnancy via Caesarean section due to maternal HELLP syndrome. The Apgar score at 1 and 5 min was 3 and 5, respectively. Neurological examination revealed moderate spastic tetraplegia, axial hypotonia, diffuse fasciculations, facial diparesis, tongue atrophy with fasciculations, scoliosis, amyotrophy, and dysarthria without cognitive impairment.

Patient 5. The patient is a 2-year-old boy, brother of patient 3 and 4, presented with 1-year history of progressive psychomotor regression and dysphagia. During pregnancy, polyhydramnios and intrauterine growth restriction was noted. The patient was born at 37 weeks of pregnancy via Caesarean section due to maternal HELLP syndrome. The Apgar score at 1 and 5 min was 7 and 9, respectively. At birth patient was normal without hypotonia or respiratory distress. His previous motor milestones were unremarkable until 1 year of age when he was able to maintain head control upright and stable sit but was never able to walk independently. Neurological examination revealed moderate spasticity in the lower limbs, brisk tendon reflexes, bilateral Babinski sign, tetraplegia with weakness of proximal and distal muscle groups, axial hypotonia, fasciculations in lower limbs, tongue atrophy, global amyotrophy, unable to sit unsupported with preserved cognition.

### Radiological findings

**Fig. 3:**
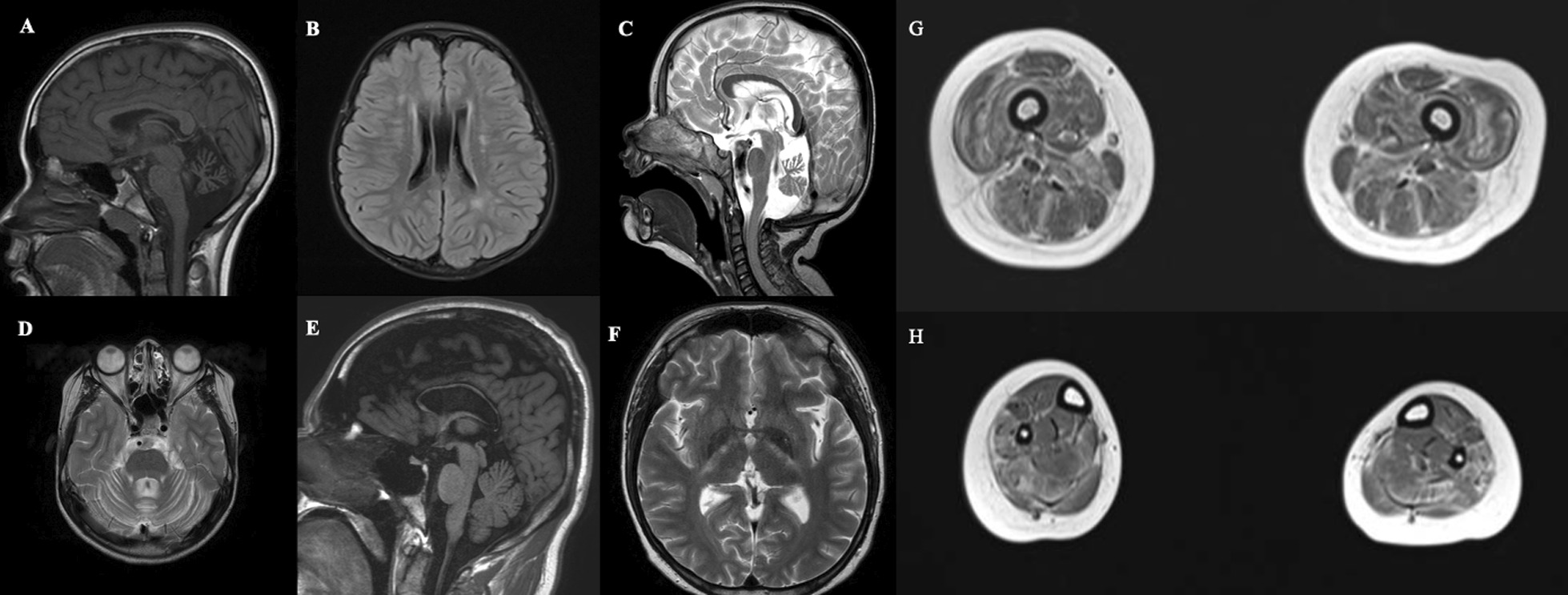
Brain and muscle MRI. **a**, **b** Sagittal T1-weighted and FLAIR images from patient 5 exhibiting cerebellar atrophy and unspecific white change abnormalities. **c**, **d** Sagittal and Axial T2-weighted images from patient 2 evidencing cerebellar atrophy with predominant involvement of superior vermis. **e**, **f** Sagittal T1-weighted and Axial T2-weighted images from patient 3 showing thin corpus callosum and bilateral corticospinal tract hyperintensity. **g**, **h** Axial T1-weighted images from thighs and legs of patient 3 exhibiting diffuse abnormal signal distributed in a “reticular pattern” trough all muscle groups and important atrophy of anterior, medial, and posterior muscle groups

The 5 patients performed brain and muscle MRI and specific changes are illustrated in Fig. 3. All five patients on muscle MRI presented images abnormalities characterized as an abnormal signal distributed in “small islands” or “reticular pattern” through all lower limb muscle groups and important atrophy of anterior, medial, and posterior muscle groups at thigh level.

In brain MRI studies, 4 patients presented cerebellar atrophy with preferential involvement of superior vermis cerebellar, 3 patients exhibited thin corpus callosum, 2 patients showed corticospinal tract hyperintensity and 2 patients presented unspecific white matter changes. All radiological findings of each patient are detailed in Table 2.

**Table 2:** Neuroimaging and neurophysiological studies

	Patient 1	Patient 2	Patient 3	Patient 4	Patient 5
*Brain MRI features*					
Cortical atrophy	Yes	No	No	Yes	No
Cerebellar atrophy	Yes	Yes	No	Yes	Yes
Thin corpus callosum	Yes	No	Yes	Yes	No
White matter changes	Yes	No	No	No	Yes
Hydrocephalus	No	No	No	No	No
Basal ganglia atrophy	No	No	Yes	Yes	No
Corticospinal tract hyperintensity	No	No	Yes	Yes	No
*Electromyography features*					
Insertional activity	Increased (C, L)	Increased (C, T, L)	Increased (C)	Increased (B, C, L)	Normal
Fasciculation	Yes (B, C, L)	Yes (B, C, L)	Yes (B, C, T, L)	Yes (B, C, T, L)	No
Fibrillation	Yes (B, C, L)	Yes (B, C, L)	Yes (B, C, T, L)	Yes (B, C, T, L)	No
positive sharp waves	Yes (B, C, L)	Yes (B, C, L)	Yes (B, C, T, L)	Yes (B, C, T, L)	No
Polyphasic motor potentials	Yes (B, C, T, L)	Yes (B, C, T, L)	Yes (B, C, T, L)	Yes (B, C, T, L)	Yes (B, C, T, L)
Amplitude motor potentials	Increased (B, C, T, L)	Increased (B, C, T, L)	Increased (B, C, T, L)	Increased (B, C, T, L)	Increased (B, C, T, L)
Duration of motor potentials	Increased (B, C, T, L)	Increased (B, C, T, L)	Increased (B, C, T, L)	Increased (B, C, T, L)	Increased (B, C, T, L)
Recruitment of motor potentials	Decreased (B, C, T, L)	Decreased (B, C, T, L)	Decreased (B, C, T, L)	Decreased (B, C, T, L)	Decreased (B, C, T, L)
Interference patterns	Rarefied (B, C, T, L)	Rarefied (B, C, T, L)	Rarefied (B, C, T, L)	Rarefied (B, C, T, L)	Rarefied (B, C, T, L)

B: Bulbar segment. C: cervical segment. T: thoracic segment. L: lumbosacral segment

## Neurophysiological features

In our cohort, all patients disclosed chronic denervation findings characterized by large-amplitude, long-duration and polyphasic motor unit action potentials in bulbar, cervical, thoracic, and lumbosacral segments, and 4 (80%) patients had also acute denervation findings characterized by fibrillation potentials, positive waves and fasciculations in at least one of the studied segments. All neurophysiological studies are summarized in Table 2.

### Genetic results

The five patients presented in this study were homozygous for the known pathogenic frameshift variant c.335dupG (p.Cys112Trpfs*11) in the *SOD1* gene that have been associated with the phenotype of Spastic Tetraplegia and Axial Hypotonia (STAHP). The parents of patients were segregating the variant in heterozygosity.

### Superoxide dismutase activity assay

**Fig. 4:**
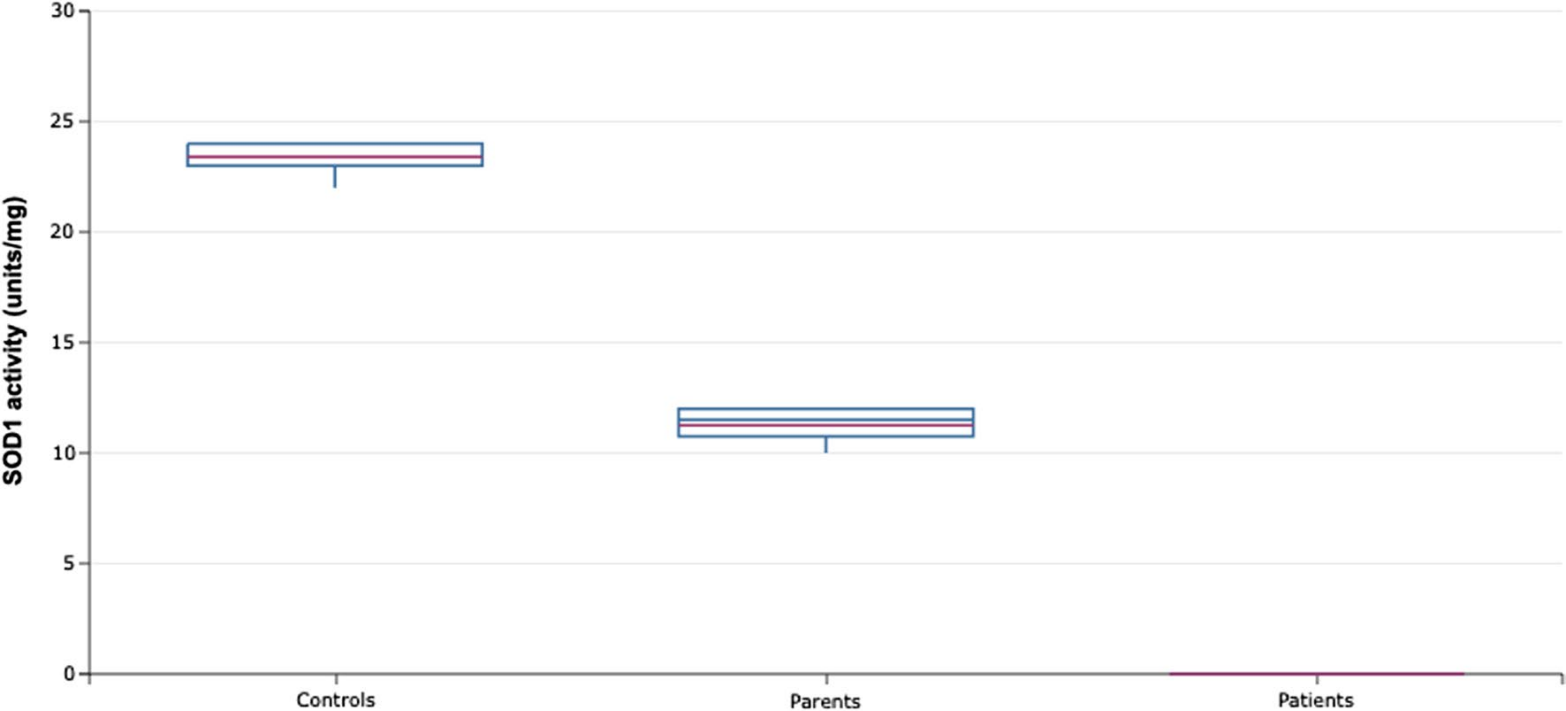
SOD1 activity. The graphic shows the SOD1 activity from erythrocytes of the five patients and their parents and five healthy controls without *SOD1* genetic variants

All patients exhibited lack of SOD1 activity in erythrocytes (< 2 units/mg), whereas the level of activity in heterozygous parents was half that of the normal level when compared to five healthy non-related controls. The values of superoxide dismutase functional activity for all patients, parents and controls are summarized in Fig. 4.

### Muscle biopsy findings

**Fig. 5:**
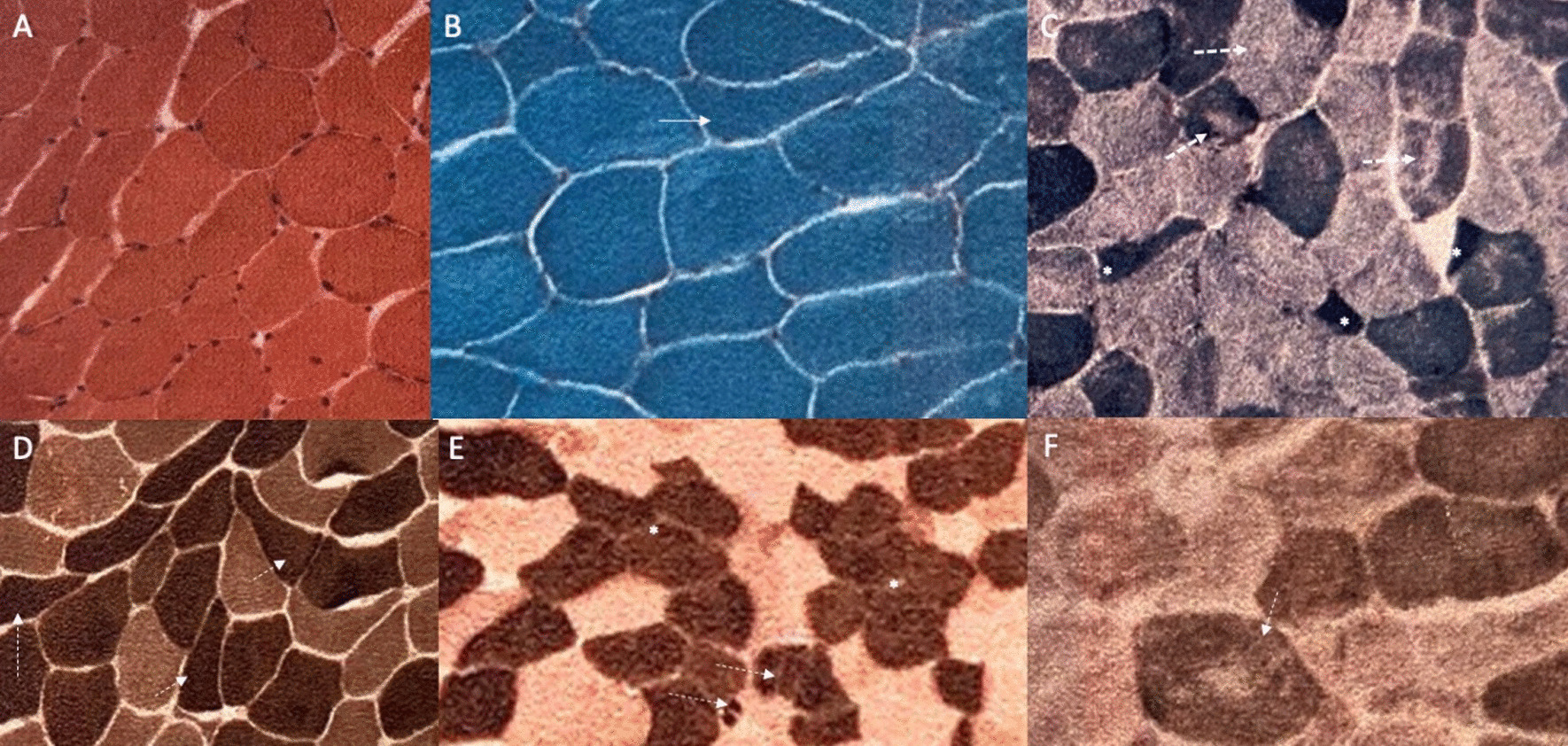
Muscle biopsy features. **a** Hematoxylin–eosin (HE) staining disclosing mild variation in muscle fiber diameter and nuclei centralization; **b** modified Gömöri trichrome staining showed angular fibers (white arrow); **c** NADH-tetrazolium reductase (NADH-TR) showed target fibers (white arrows) and angular fibers (asterisk); **d** ATPase stain at pH 4.6 and **e** ATPase stain at pH 9.4 showed angular fibers (white arrows) and tendency to type grouping formation (asterisks); **f** Cytochrome C-oxidase (COX) reaction disclosed focal central areas with reduced staining

All patients performed muscle biopsy that showed pathological findings suggestive of neurogenic changes such as increased fiber size variability with angulated atrophic fibers rounded by hypertrophic fibers, increased number of type 2 fibers but without type grouping formation and without endomysium changes and some “target-fibers” observed in NADH-TR reaction (Fig. 5).

### Laboratory analysis

All patients presented with hiperCKemia ranging between 400 and 780 UI/L, and had normal values for serum lactate, acylcarnitine profile and organic acids in urine. The CPK levels for each patient are detailed in Table 1.

### Functional assessment

In this cohort, all patients required gastrostomy for nutritional management, and all are dependent on mechanical ventilation, with 3 patients having tracheostomy and use of invasive mechanical ventilation 24 h per day, and 2 patients on non-invasive mechanical ventilation for at least 18 h per day. None of the patients are able to walk or sit in unassisted fashion. All patients presented a score less than 10 points at HINE-2 and the maximum score at CHOP-INTEND was 18 points. The detailed results of functional assessment are summarized in Table 3.

**Table 3:** Functional assessment data

	Patient 1	Patient 2	Patient 3	Patient 4	Patient 5
*HINE-2*					
Head control	1	1	2	1	1
Sitting	1	2	2	1	1
Voluntary grasp	3	3	3	3	3
Ability to kick in supine	0	0	1	1	1
Rolling	0	1	0	0	1
Crawling	0	0	0	0	0
Standing	0	0	0	0	0
Walking	0	0	0	0	0
Total	5	7	8	6	7
*CHOP INTEND*					
Total	8	18	12	14	14
HFMSE					
Total	3	7	5	5	3

HFMSE: Hammersmith Functional Motor Scale for SMA. HINE-2: Hammersmith Infant Neurological Examination-Sect. 2-Motor Milestones. CHOP INTEND: Children’s Hospital of Philadelphia Infant Test of Neuromuscular Disorder

## Discussion

Amyotrophic Lateral Sclerosis (ALS) is a progressive neurodegenerative disorder characterized by the degeneration of upper and lower motor neurons and clinically characterized by progressive motor deficits, and varying progression of speech deterioration with heterogenous cognitive and behavioral changes [13].

Traditionally, ALS is classified as sporadic ALS, that accounts for 90% cases, and familial ALS, when there is a context of more than one affected family member (relative) in the first or second generation with a similar disease presentation of the index patients, or with ALS-FTD presentation and that so far accounts for up to 10% of cases in large series [4, 14]. Until 2014, 22 genes were implicated as causative factor in genetically determined ALS correlated with mutations in these genes, accounting for about two-thirds of all familial cases, and approximately 10% of sporadic ALS. Recently, more genes have been associated to familial ALS due to improvements in genetic sequencing technologies, and a growing number of patients have been identified with genetically determined ALS genetic leading to an expansion of disease phenotype spectrum, and creating a great challenge in genetic counseling, but offering the opportunity to better understand the pathophysiology that is key for the development of new disease-modifying therapies [4, 14].

The pathophysiology of ALS is poorly understood and can rise as a consequence of multiple pathophysiological mechanisms and cellular dysfunctions, including protein misfolding and aggregation, altered RNA processing, defects in axonal transport, abnormal metabolism and accumulation of reactive oxygen species, mitochondrial dysfunctions, microglial and neuroinflammatory mechanisms, disturbances of autophagy, ubiquitin–proteasome system abnormalities and primary and secondary ion channel defects [4, 14–16].

Since 1993, when *SOD1* gene variants were first associated to familial ALS, more than 150 variants have been described and the mechanism of SOD1 protein dysfunction in the pathophysiology of disease was partially elucidate, and new genetic therapies are emerging from recent clinical trials and are on the verge of being used in clinical practice as innovative disease-modifying therapy for the treatment of ALS [17, 18].

The exact mechanism on how *SOD1* variants leads to ALS is yet incompletely understood, with current knowledge indicating that the pathogenic variants produce a destabilization of SOD1 structure protein, with a gain of function mechanism that leads to an increase oxidative activity with excessive production of hydrogen peroxide, and raise in protein–protein interactions predisposing to increased aggregation, dimer destabilization, and oligomerization resulting in abnormal axonal transport, microglia activation, increased apoptosis, mitochondrial dysfunction and oxidative stress that at last plays a crucial roles in motor dysfunction [2, 4, 19–22].

Juvenile Amyotrophic Lateral Sclerosis (JALS) is empirically classified as a phenotypic variant of ALS with onset of disease before 25 years of age and having a slower disease progression with prolonged survival compared to adult-onset ALS. JALS has a rare frequency with an estimated prevalence of 1 case per 1.000.000 and generally associated with autosomal recessive or autosomal dominant inheritance with highly penetrant genetic variants in causative genes as *ALS2*, *SPG11*, *SIGMAR1*, *SETX*, *SOD1*, *UBQLN2* and *FUS* [23–25].

The diagnosis of ALS and their variants such as JALS can be an arduous and labored challenge and since 1994 many diagnostic criteria were made and refined from the El Escorial to Gold Coast with the latter and current criteria for diagnosis of ALS is based in three statements: 1) progressive motor impairment documented by history or repeated clinical assessment, preceded by normal motor function, and; 2) presence of upper and lower motor neuron dysfunction in at least 1 body region (with upper and lower motor neuron dysfunction noted in the same body region if only one body region is involved) or lower motor neuron dysfunction in at least 2 body regions, and; 3) additional investigations excluding other disease process [26, 27]. The evidence of upper motor neuron dysfunction implies at least one of the following clinical signs: (a) increased deep tendon reflexes, including the presence of a reflex in a clinically weak and wasted muscle, or spread to adjacent muscles; (b) presence of pathological reflexes, including Hoffman sign, Babinski sign, crossed adductor reflex, or snout reflex; (c) increase in velocity-dependent tone (spasticity); (d) slowed, poorly coordinated voluntary movement, not attributable to weakness of lower motor origin or Parkinsonian features [27]. Lower motor neuron dysfunction requires either: (a) clinical evidence of muscle weakness and muscle wasting; or (b) electromyography abnormalities that must include both evidence of chronic and ongoing denervation [27]. Supportive evidence of lower motor neuron dysfunction can be obtained by ultrasound detection of fasciculations from multiple muscles, and supportive evidence of upper motor neuron dysfunction can be derived from transcranial magnetic stimulation studies of the central motor nervous system, MRI, and neurofilament levels [28, 29].

A critical review of the case described by Andersen et al. who reported on a patient with clear signs of upper motor neuron dysfunction, and some evidence of lower motor neuron dysfunction revealed by fasciculations and myokymia in electromyography, that however are insufficient to confirm a lower motor neuron involvement according to Gold Coast Criteria for ALS. Moreover, a clinical follow-up was lacking, to detect progression of disease, and there was no serial electromyography evaluation to detect further lower motor neuron dysfunction.

The case reported by Park et al. refers to a six-year-old patient who presented with significant upper motor neuron dysfunction and more substantial evidence of lower motor neuron dysfunction, with multiple fasciculations of the right deltoid, right extensor digitorum, vastus medialis and tibialis anterior muscles on both sides described by muscle ultrasound and confirmed by the detection of fasciculations on electromyography without any additional neurogenic changes. Again, these exams were insufficient to establish a definitive diagnosis of ALS according to Gold Coast criteria considering the lack of long-term clinical follow-up and absence of other electromyography studies that are useful to confirm a lower motor neuron dysfunction overtime [6].

Our study presents a series of five patients that typify a progressive motor. neuron disorder with undoubtful upper and lower motor neuron involvement demonstrated by clinical examination, and corroborated by neurophysiological and muscle pathological studies that fulfills all the recently proposed diagnostic criteria for ALS with the same homozygous pathogenic variant in *SOD1* gene reported by Andersen et al., and Park et al. allowing us to conclude that the cases presented by these authors, and the new additional patients reported in this study represent a very early infantile-onset ALS. Regarding parental carriers that are heterozygous for the variant c.335dupG (p.Cys112Trpfs*11) in the *SOD1* gene, we still cannot say anything about the future risk of developing a neurodegenerative disease, and they need long-term clinical follow-up to early detect neurological manifestations related to ALS. The absence of neurological manifestations in the parents described in the literature, and in our experience can be related to recessive mechanism of transmission, or a low penetrance for the variant c.335dupG like other variants described in *SOD1* gene [30–33].

The exact pathophysiological mechanism that the homozygous c.335dupG leads to the ALS phenotype observed in our cohort and the STAHP described in the literature is not completely understood. However, we can hypothesize that complete loss of function of SOD1 enzyme can produce an increased vulnerability to oxidative stress with important mitochondrial dysfunction as demonstrated by animal models like *Sod1*^−/−^ mice that have been associated with neuromuscular, neuronal, and extra-neuronal phenotypes [34].

## Conclusions

SOD1 protein has a critical role in the function and homeostasis of motor neurons and SOD1 deficiency is related to very early-onset juvenile ALS as described in this article. This unusual “new” phenotype of infantile neurodegenerative disorder (STAHP), which is similar to ALS, and juvenile motor neuron disease, should be always recalled in the differential diagnosis of neurodegenerative disorders in childhood with progressive involvement of motor neurons [35].

## Data Availability

The datasets are not publicly available due to individual privacy reasons (patients’ confidentiality).

## Abbreviations

ALS: Amyotrophic lateral sclerosis
CHOP-INTEND: Children’s hospital of philadelphia infant test of neuromuscular disorder
CPK: Serum creatine kinase
EMG: Electromyography
FTD: Frontotemporal dementia
HFMSE: Expanded hammersmith functional motor scale for SMA
HINE-2: Hammersmith infant neurological examination-Section 2-motor milestones
JALS: Juvenile amyotrophic lateral sclerosis
MRI: Magnetic resonance imaging
NCS: Nerve conduction studies
SODs: Superoxide dismutase enzymes
SOD1: Human Cu–Zn superoxide dismutase 1
STAHP: Spastic tetraplegia with axial hypotonia
WES: Whole exome sequencing
